# PARP inhibitors protect against sex- and AAG-dependent alkylation-induced neural degeneration

**DOI:** 10.18632/oncotarget.19844

**Published:** 2017-08-03

**Authors:** Mariacarmela Allocca, Joshua J. Corrigan, Kimberly R. Fake, Jennifer A. Calvo, Leona D. Samson

**Affiliations:** ^1^ Department of Biological Engineering, Massachusetts Institute of Technology, Cambridge, MA 02139, USA; ^2^ Department of Biology, Massachusetts Institute of Technology, Cambridge, MA 02139, USA; ^3^ Center for Environmental Health Sciences, Massachusetts Institute of Technology, Cambridge, MA 02139, USA; ^4^ David H. Koch Institute for Integrative Cancer Research, Massachusetts Institute of Technology, Cambridge, MA 02139, USA

**Keywords:** alkylating agents, PARP1, PARP inhibitors, AAG/MPG, retinal and cerebellar degeneration

## Abstract

Alkylating agents are commonly used to treat cancer. Although base excision repair (BER) is a major pathway for repairing DNA alkylation damage, under certain conditions, the initiation of BER produces toxic repair intermediates that damage healthy tissues. The initiation of BER by the alkyladenine DNA glycosylase (AAG, a.k.a. MPG) can mediate alkylation-induced cytotoxicity in specific cells in the retina and cerebellum of male mice. Cytotoxicity in both wild-type and *Aag*-transgenic (*AagTg*) mice is abrogated in the absence of Poly(ADP-ribose) polymerase-1 (PARP1). Here, we tested whether PARP inhibitors can also prevent alkylation-induced retinal and cerebellar degeneration in male and female WT and *AagTg* mice. Importantly, we found that WT mice display sex-dependent alkylation-induced retinal damage (but not cerebellar damage), with WT males being more sensitive than females. Accordingly, estradiol treatment protects males against alkylation-induced retinal degeneration. In *AagTg* male and female mice, the alkylation-induced tissue damage in both the retina and cerebellum is exacerbated and the sex difference in the retina is abolished. PARP inhibitors, much like *Parp1* gene deletion, protect against alkylation-induced AAG-dependent neuronal degeneration in WT and *AagTg* mice, regardless of the gender, but their efficacy in preventing alkylation-induced neuronal degeneration depends on PARP inhibitor characteristics and doses. The recent surge in the use of PARP inhibitors in combination with cancer chemotherapeutic alkylating agents might represent a powerful tool for obtaining increased therapeutic efficacy while avoiding the collateral effects of alkylating agents in healthy tissues.

## INTRODUCTION

DNA damage is continually induced by environmental agents and chemically reactive byproducts of normal cellular metabolism. Moreover, DNA damage is often deliberately induced during the course of cancer chemotherapy. One class of commonly-utilized chemotherapy agents is alkylating agents, a broad group of compounds that add alkyl groups to DNA. Alkylating agents generate numerous types of alkylated DNA base lesions, including *O*^6^-methylguanine (*O*^6^meG), 7-methylguanine (7meG) and 3-methyladenine (3meA). The effectiveness of alkylators as cancer chemotherapeutic agents relies on the induction of cell death in rapidly dividing tumor cells by toxic DNA lesions that interfere with DNA replication and RNA transcription. DNA repair pathways have evolved to cope with recurring DNA damage and, for the most part, provide protection in healthy tissues. The base excision repair (BER) pathway repairs a variety of alkylated DNA bases [1, 2], and the AAG DNA glycosylase (a.k.a. MPG) initiates BER by recognizing and excising 7meG and 3meA. Following base excision, an apurinic/apyrimidinic endonuclease (APE1) hydrolyzes the phosphodiester backbone at the abasic site, generating a single-stranded DNA break (SSB) with 3’OH and 5’deoxyribose-5-phosphate (5’dRP) termini. DNA polymerase Δ (Pol Δ) removes the 5’dRP terminus and conducts single-nucleotide gap filling synthesis. BER is completed upon ligation of the nicked DNA by DNA Ligase I or the Xrcc1/Ligase IIIα complex. Poly(ADP-ribose) polymerase 1 (PARP1, a.k.a. ADP-Ribosyltransferase1/ADPRT1 or ADP-Ribosyltransferase Diphtheria Toxin-Like 1/ARTD1) is a multi-functional protein that mediates several cellular processes and plays an important role in BER [3–12]. PARP1 acts as a SSB sensor, and, upon binding a SSB containing a 5’dRP terminus, is activated to use NAD^+^ to catalyze the addition of long branched polymers of ADP-ribose (PAR) to several nuclear proteins, including itself, DNA polymerases, DNA ligases, transcription factors and histones [9, 13, 14]. PARylation of histones, PARP1, and chromatin remodeling enzymes serves to relax chromatin, allowing DNA repair proteins access to DNA damage [15–17]. Moreover, PARP1 auto-modification is thought to recruit XRCC1, the BER scaffold protein that facilitates the formation of a complex of BER enzymes, including Pol Δ and DNA Ligase III [18–20]. With the completion of repair, PARP1 dissociates from the DNA and PAR is rapidly cleaved, primarily by PAR glycohydrolase [21]. Taken together, these aspects of PARP1’s function serve to facilitate BER, allowing cells to recover from DNA damage.

Although BER can efficiently repair DNA alkylation damage in most cells, in certain cell types, the initiation of BER can generate toxic repair intermediates that cause damage to healthy tissues. Both SSBs and AP sites exert their toxicity as a function of blocking transcription and replication [22] and by generating mutations via translesion DNA synthesis [23–26]. Further, large numbers of SSBs can indirectly induce toxicity through hyperactivation of PARP1. Indeed, PARP1 can act as a cell death mediator [27, 28]; upon excessive DNA damage, PARP1 hyperactivation vastly increases NAD^+^ consumption, resulting in depletion of both NAD^+^ and ATP, such that cells succumb to bioenergetic failure and necrotic cell death [27, 29, 30]. Independent of NAD^+^/ATP depletion, the PAR polymer inhibits the hexokinase 1, resulting in the block of glycolysis with consequent energy collapse and cell death [31]. The PAR polymer can also promote cell death by facilitating translocation of the apoptosis inducing factor (AIF) from mitochondria to the nucleus, resulting in chromatin condensation, caspase-independent DNA degradation, and ultimately cell death [32–34]. Therefore, although BER is essential for the repair of many different types of DNA damage, it must be carefully regulated to avoid any imbalance and consequent accumulation of toxic BER intermediates.

Recently, using mouse genetic models, we demonstrated the importance of both AAG and PARP1 in modulating *in vivo* alkylation toxicity in healthy tissues. The alkylating agent, methyl methanesulfonate (MMS), induces tissue damage in a specific subset of tissues, including retina and cerebellum [35, 36], in WT male mice. Modest increases in AAG activity in a transgenic mouse model (*AagTg* mice) increase (rather than decrease) susceptibility to the alkylating agent for both whole-animal survival and for tissue damage [35, 36]. In the absence of AAG activity, these tissues are remarkably refractory to MMS-induced cell death. The AAG-mediated alkylation sensitivity in the retina and cerebellum, for both wild-type (WT) and *AagTg* mice, is entirely PARP1-dependent, being wholly prevented in the absence of PARP1 resulting from a null mutation in the *Parp1* gene.

Several PARP inhibitors have been developed, with some in advanced clinical trials for the treatment of various tumors, either alone or in combination with other chemotherapeutics [37, 38]. These include Veliparib and Olaparib, whose structures contain a nicotinamide moiety that competes with NAD^+^ for PARP binding, making them efficient catalytic inhibitors of PARP. In addition to catalytic inhibition, the potency of PARP inhibitors also depends on their DNA trapping ability [39]. It turns out that PARP inhibitors can trap PARP1 on DNA, forming complexes that interfere with replication and transcription [39–45]. In the presence of PARP inhibitors, PARP1 still binds to SSBs, but auto-PARylation is prevented; inhibited PARP1 thus becomes trapped on the BER intermediates. The trapped/inhibited PARP1 complex eventually leads to stalled replication forks, accumulation of double-strand breaks (DSB) and cell death. Hence, PARP inhibitors can have opposite effects: (i) promote cell death by diminishing BER or by trapping PARP1 on DNA, leading to the induction of DSBs; (ii) prevent cell death by avoiding PARP1 hyperactivation and NAD^+^/ATP depletion.

Here, we tested whether the MMS-induced neuronal degeneration in WT and *AagTg* mice would be inhibited or exacerbated by the PARP inhibitors, Veliparib and Olpaparib. Since gender differences have been reported in response to *Parp1* deletion and PARP inhibition [46–50], we included both male and female mice in this study. Importantly, we found that WT retinal photoreceptor tissue (but not cerebellar tissue) displays sex-dependent alkylation-induced damage, with WT males being more sensitive than females; this sex difference in alkylation-induced tissue damage is abolished when AAG levels are increased above physiological levels in *AagTg* mice. Moreover, we show that Olaparib and Veliparib, like the *Parp1* gene deletion, protect against MMS-induced AAG-dependent retinal and cerebellar damage in both WT and *AagTg* mice, regardless of gender. Overall, our findings further underscore the recent push for investigation and understanding of sex differences in diseases where pathophysiological mechanisms involve PARP1. Moreover, our data are of particular relevance, given the wide range of AAG activity in the human population that we and others have reported [35, 51, 52]. Neuronal degeneration may represent potential collateral damage when alkylating agents are used for chemotherapy, especially for patients with high AAG activity. The recent surge in the use of PARP inhibitors in combination with alkylating agents [37, 38] may represent a powerful tool for obtaining better therapeutic efficacy in cancer treatment, while avoiding the collateral effects of alkylation damage in healthy tissues.

## RESULTS

### Treatment with PARP inhibitors protects against MMS-induced retinal degeneration

We have shown that the AAG DNA glycosylase, an enzyme that initiates BER, drives alkylation-induced cytotoxicity in retinal photoreceptors in WT mice and in a transgenic mouse model (*AagTg*) expressing increased levels of AAG [35, 36]. Compared to WT mice, retinal photoreceptors display increased alkylation sensitivity in *AagTg* mice and remarkable resistance in *Aag^−/−^* mice [35]. This AAG-mediated alkylation sensitivity, for both WT and *AagTg* mice, is entirely PARP1-dependent, being completely suppressed by *Parp1* gene deletion [35]. To determine whether drug-mediated PARP inhibition also protects against alkylation-induced retinal degeneration, we treated WT and *AagTg* mice with either Veliparib (10 mg/kg) or Olaparib (50 mg/kg) 1 hour prior to MMS injection (75 mg/kg), and then analyzed retinas 7 days (d) post-MMS. Gender differences have been reported in response to both *Parp1* deletion and PARP inhibition [46–50]; moreover, differences between *Parp1* deletion versus PARP inhibition have also been described [53, 54]. We, therefore, grouped the results by gender and compared the effects of PARP inhibition in MMS-treated WT, *AagTg, Parp1^−/−^* and *AagTg/Parp1^−/−^* mice.

**Figure 1:**
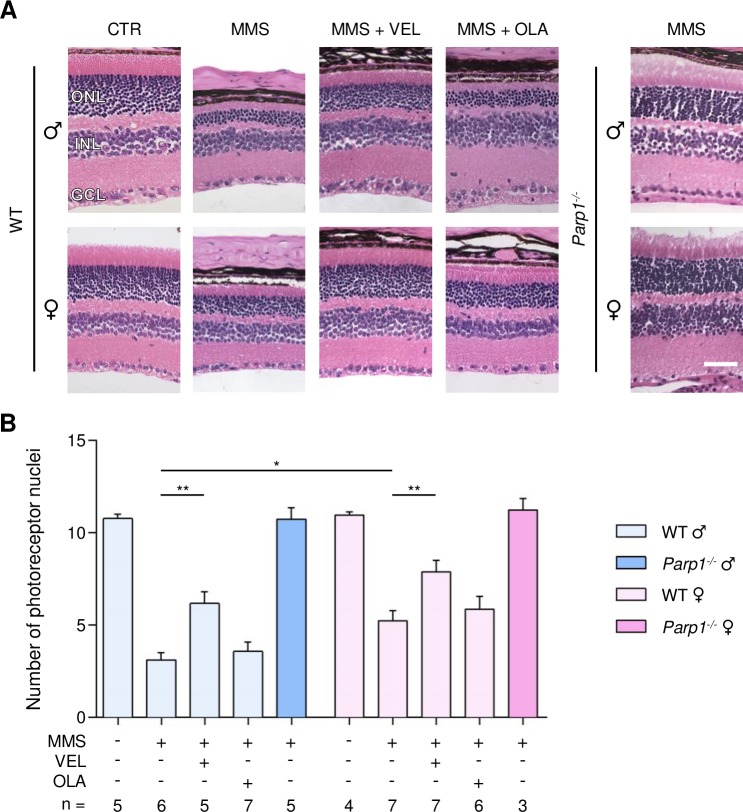
Treatment with PARP inhibitors protects WT mice against sex- and AAG-dependent MMS-induced retinal degeneration **(A)** Representative H&E-stained images of retinas from WT mice and *Parp1^−/−^* mice 7 days after MMS (75 mg/kg) and PARP inhibitor (VEL, Veliparib 10 mg/kg; OLA, Olaparib 50 mg/kg) treatment, as indicated. PARP inhibitors were administered to WT mice 1 hour prior to MMS treatment. Magnification is 200X (scale bar 50 μm); ONL, outer nuclear layer; INL, inner nuclear layer; GCL, ganglion cell layer; CTR, untreated control mice; ♂, males; ♀, females. **(B)** Quantification of rows of photoreceptor nuclei in the outer nuclear layer of WT and *Parp1^−/−^* mice 7 days after MMS (75 mg/kg) and PARP inhibitors (VEL, Veliparib 10 mg/kg; OLA, Olaparib 50 mg/kg) treatment, as indicated. *, p < 0.05; **, P < 0.01; ♂, males; ♀, females.

MMS induces selective degeneration of the photoreceptor cells located in the outer nuclear layer (ONL) of the retina [35, 36]. Retinal degeneration was quantified by counting the rows of photoreceptor nuclei in the ONL on stained histological sections, as described in the Materials and Methods. For all the genotypes, untreated male and female mice have about 11 rows of photoreceptor nuclei. MMS-treated WT males had significantly fewer rows of photoreceptor nuclei, as described previously (Figure 1) [35, 36]; it should be noted that all our previous studies used male mice exclusively. Here, we found that WT female mice were partially protected from MMS-induced retinal degeneration compared to male mice (5.2 ± 0.5 rows in females versus 3.1 ± 0.3 rows in males, p = 0.01, Figure 1). Veliparib treatment partially protected WT mice from MMS-induced retinal degeneration regardless of gender (6.2 ± 0.6 rows in males and 7.8 ± 0.6 in females, Figure 1). In contrast, Olaparib treatment did not protect either male or female WT mice (3.5 ± 0.5, males; 5.8 ± 0.6, females; Figure 1). Veliparib or Olaparib treatment itself induced no retinal damage (Supplementary Figures 1 and 2). *Parp1* gene deletion completely rescued the MMS-induced retinal degeneration in male mice, as seen previously [35], and in female *Parp1^−/−^* mice as well (Figure 1).

**Figure 2:**
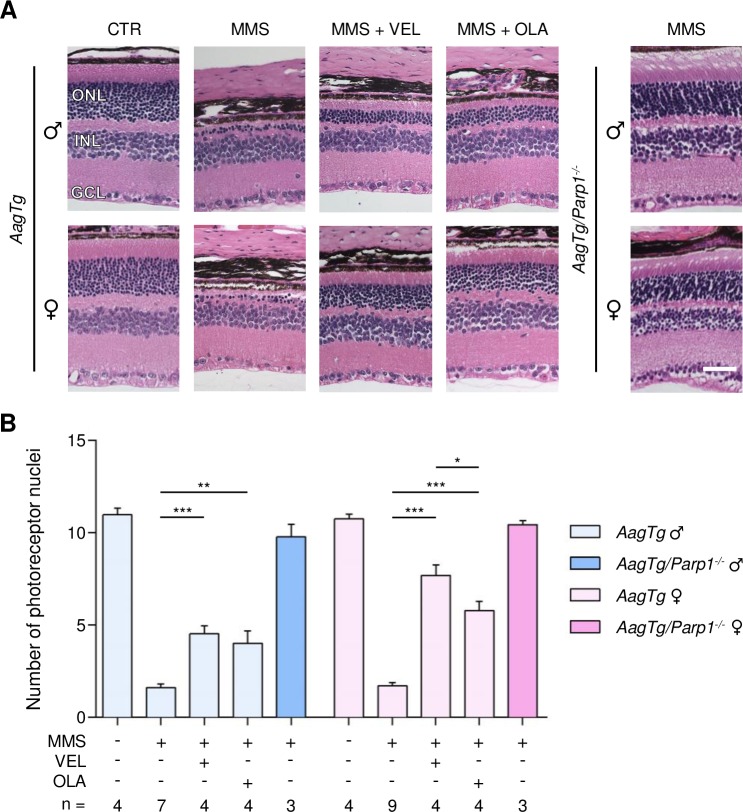
Treatment with PARP inhibitors protects *AagTg* mice against AAG-dependent MMS-induced retinal degeneration **(A)** Representative H&E-stained images of retinas from *AagTg* and *AagTg/Parp1^−/−^* mice 7 days after MMS (75 mg/kg) and PARP inhibitor (VEL, Veliparib 10 mg/kg; OLA, Olaparib 50 mg/kg) treatment, as indicated. PARP inhibitors were administered to *AagTg* mice 1 hour prior to MMS treatment. Magnification is 200X (scale bar 50 μm); ONL, outer nuclear layer; INL, inner nuclear layer; GCL, ganglion cell layer; CTR, untreated control mice; ♂, males; ♀, females. **(B)** Quantification of rows of photoreceptor nuclei in the outer nuclear layer of *AagTg* and *AagTg/Parp1^−/−^* mice 7 days after MMS (75 mg/kg) and PARP inhibitor (VEL, Veliparib 10 mg/kg; OLA, Olaparib 50 mg/kg) treatment, as indicated. *, p < 0.05; **, P < 0.01; ***, p < 0.001; ♂, males; ♀, females.

By comparison with WT, *AagTg* mice showed extreme retinal sensitivity to MMS treatment, as described previously [35, 36] (Figure 2). Importantly, no difference in MMS sensitivity was observed between male and female *AagTg* mice (1.6 ± 0.2 versus 1.7 ± 0.1, Figure 2). Veliparib treatment partially protected *AagTg* mice from MMS-induced retinal degeneration, regardless of gender (Figure 2). Notably, protection in *AagTg* female mice was more robust than in *AagTg* male (7.6 ± 0.5 in females versus 4.5 ± 0.4 in males, p < 0.0001). In contrast to what we observed in WT, Olaparib treatment partially protected both male and female *AagTg* mice (4.0 ± 0.6, p = 0.002 and 5.7 ± 0.4, p < 0.0001, respectively). As with Veliparib, protection in Olaparib-treated *AagTg* female mice was slightly more robust than in Olaparib-treated *AagTg* males (5.8 ± 0.4 in females versus 4.0 ± 0.6 in males, p = 0.07; Figure 2). Greater protection was observed in Veliparib-treated *AagTg* female mice as compared with Olaparib-treated *AagTg* female mice (7.6 ± 0.5 versus 5.7 ± 0.4, p = 0.045; Figure 2). *Parp1* gene deletion in *AagTg* mice (*Parp1^−/−^/AagTg*) completely rescued the MMS-induced retinal degeneration, regardless of gender (Figure 2).

Additional groups of mice were treated with PARP inhibitors 1 hour pre- and 24 hours post-MMS injection. Combined pre- and post-MMS treatments with PARP inhibitors (represented as x2 in Supplementary Figures 1 and 2) showed results similar to those obtained with a single pre-MMS treatment, and no additional retinal protection was observed (Supplementary Figures 1 and 2). Instead, retinal protection was significantly reduced in *AagTg* females treated with Veliparib pre- and post-MMS, as compared to *AagTg* females that received one dose of Veliparib pre-MMS (5.0 ± 0.7 vs 7.7 ± 0.5, p < 0.05).

Our findings demonstrate the following: (i) the retina displays sex-dependent alkylation-induced degeneration with males being more susceptible than females; (ii) the difference in response between males and females is abolished when AAG levels are increased; (iii) PARP inhibitors protect against MMS-induced AAG-dependent retinal degeneration but, unlike with the *Parp1* deletion, the degree of PARP inhibitor-mediated protection depends on the specific PARP inhibitor used, AAG levels and mouse gender.

MMS treatment causes significant weight loss in both male and female *AagTg* mice at 24 hours post-treatment, regardless of gender, an effect that is not observed in WT mice (Supplementary Figure 3) [35, 55]. Indeed, 24 hours post-MMS treatment, the body weights (BW) of male and female *AagTg* mice were 87.0 ± 0.9% and 89.7 ± 1.4% of their pre-MMS BW, respectively. Treatment with Veliparib partially protected both male (BW 91.2 ± 0.9%, p = 0.014) and female (BW 94.9 ± 1.1%, p = 0.014) *AagTg* mice from this weight loss, while Olaparib significantly protected male (BW 90.9 ± 0.5%, p = 0.001), but not female (91.3 ± 1.4%, p = 0.46), *AagTg* mice. These data further support the protective role of PARP inhibitors against MMS-induced tissue damage.

### Estradiol protects wild-type male mice against MMS-induced retinal degeneration

**Figure 3:**
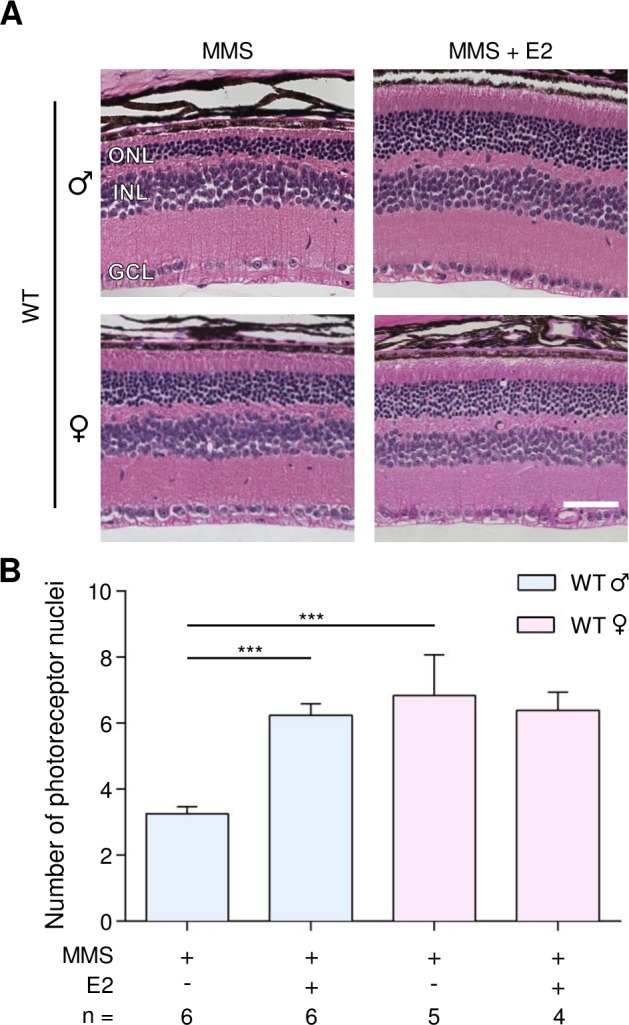
Estradiol protects WT male mice from MMS-induced retinal degeneration **(A)** Representative H&E-stained images of retinas from WT male and female mice treated with 17-Δ estradiol (E2, 50 μg/kg/day) and/or MMS (75 mg/kg), as indicated. E2 was injected 2 hours before MMS injection and daily post-MMS injection. Retinas were harvested 7 days post-MMS injection. Magnification is 200X (scale bar 50 μm); ONL, outer nuclear layer; INL, inner nuclear layer; GCL, ganglion cell layer; ♂, males; ♀, females. **(B)** Quantification of rows of photoreceptor nuclei in the outer nuclear layer of WT male and female mice, treated or not treated with E2 (50 μg/kg/day), 7 days post-MMS (75 mg/kg). ***, p < 0.001; ♂, males; ♀, females.

We have shown that WT female mice are partially protected against MMS-induced retinal degeneration, as compared to male mice. We therefore determined whether estrogen treatment in male mice could protect against MMS-induced retinal degeneration. We treated male and female mice with MMS and delivered daily intraperitoneal injections of 17-β estradiol (E2, 50 μg/kg). The first dose of E2 was given 2 hours before the single MMS injection, and tissues were collected 7 days post-MMS injection. As shown in Figure 3, the photoreceptors of male mice are more sensitive to MMS than those of female mice (3.3 ± 0.2 rows in males versus 6.8 ± 0.5 rows in females, p = 0.0001, Figure 3), corroborating the data shown in Figure 1. Figure 3 also shows that E2 protected against MMS-induced retinal degeneration in male mice, but offered no further protection in female mice (6.2 ± 0.3 rows in males; 6.4 ± 0.5 in females; Figure 3). The sexual dimorphism observed in the retina after MMS treatment is not due to sex-related differences in *Aag* expression level (Supplementary Figure 4). Our findings suggest a protective role for estrogen against alkylation-induced retinal degeneration in female mice.

### Treatment with PARP inhibitors protects against MMS-induced cerebellar degeneration

**Figure 4:**
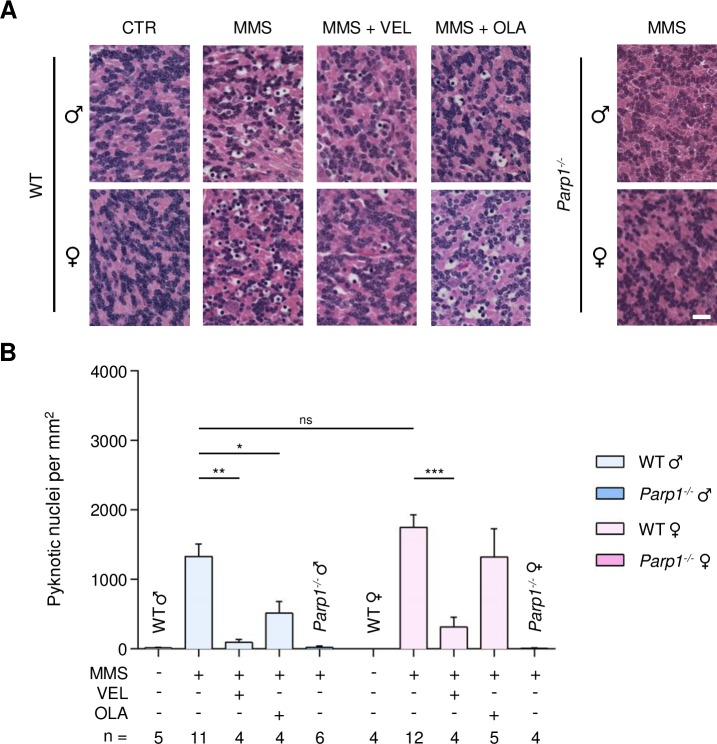
Treatment with PARP inhibitors protects WT mice against AAG-dependent MMS-induced cerebellar degeneration **(A)** Representative H&E-stained images of cerebellar granular cells from WT and *Parp1^−/−^* mice 6 hours after MMS (75 mg/kg) treatment. PARP inhibitors (VEL, Veliparib 10 mg/kg; OLA, Olaparib, 50 mg/kg) were administered to WT mice 1 hour prior MMS treatment, as indicated. Magnification is 600X (scale bar 20 μm); CTR, untreated control mice; ♂, males; ♀, females. **(B)** Quantification of pyknotic nuclei/mm^2^ in WT and *Parp1^−/−^* mice after MMS and PARP inhibitor (VEL, Veliparib 10 mg/kg; OLA, Olaparib 50 mg/kg) treatment, as indicated. *, p < 0.05; **, P < 0.01; ***, p < 0.001; ns, not statistically significant (p = 0.11); ♂, males; ♀, females.

**Figure 5:**
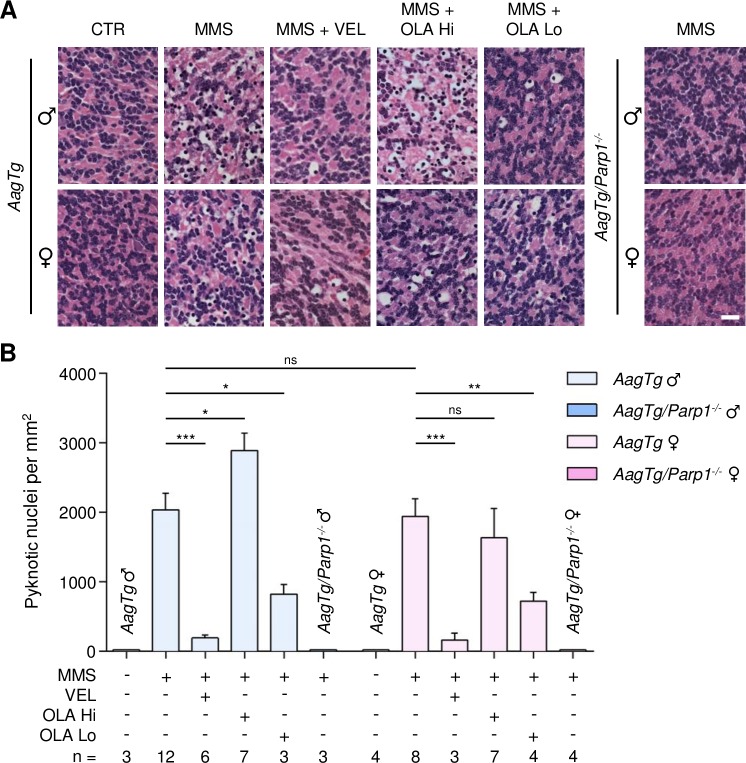
Treatment with PARP inhibitors protects *AagTg* mice against AAG-dependent MMS-induced cerebellar degeneration **(A)** Representative H&E-stained images of cerebellar granular cells from *AagTg* and *AagTg/Parp1^−/−^* mice 6 hours after MMS (75 mg/kg) treatment. PARP inhibitors (VEL, Veliparib 10 mg/kg; OLA Hi, Olaparib 50 mg/kg; OLA Lo, Olaparib 1 mg/kg) were administered to *AagTg* mice 1 hour prior MMS treatment. Magnification is 600X (scale bar 20 μm); CTR, untreated control mice; ♂, males; ♀, females. **(B)** Quantification of pyknotic nuclei/mm^2^ in *AagTg* and *AagTg/Parp1^−/−^* mice after MMS and PARP inhibitor (VEL, Veliparib 10 mg/kg; OLA Hi, Olaparib 50 mg/kg, OLA Lo, Olaparib 1 mg/kg) treatment, as indicated. *, p < 0.05; **, P < 0.01; ***, p < 0.001; ns, not statistically significant; ♂, males; ♀, females.

MMS induces AAG-dependent cell death in cerebellar granule cells, which comprise 99% of the granular layer of the cerebellum. This cerebellar degeneration is completely suppressed by *Parp1* gene deletion [35]. To determine whether PARP inhibition protects against alkylation-induced cerebellar degeneration, we administered Veliparib (10 mg/kg) or Olaparib (50 mg/kg) to both WT and *AagTg* mice 1 hour prior to MMS-injection. Due to the dramatically higher MMS sensitivity of *AagTg* mice compared to WT mice and based on our previous published results [35], in order to induce the degeneration phenotype such that one could accurately quantify the cerebellar degeneration, we used different MMS doses for each genotype: 150 mg/kg and 60 mg/kg in WT and *AagTg* mice, respectively. Cerebella were collected 6 hours post-MMS injection. Cerebellar degeneration was then quantified by counting the number of pyknotic nuclei in the granular layer of the cerebellum, as described in the Materials and Methods. In untreated mice, no pyknotic nuclei were observed (Figures 4 and 5). However, 6 hours post-MMS, we observed severe lesions containing numerous pyknotic nuclei surrounded by white space (edema) in WT mice (Figure 4), as shown previously [35]. The number of MMS-induced pyknotic nuclei was similar in male and female WT mice (1332 ± 179 in males; 1753 ± 175 in females, p = 0.11). Veliparib treatment (10 mg/kg) almost completely rescued WT mice, regardless of sex (Figure 4). Conversely, Olaparib, at the dose of 50 mg/kg, was able to partially protect male, but not female, WT mice (514 ± 171 in males and 1325 ± 405 in females; Figure 4). Veliparib or Olaparib treatment itself did not induce any cerebellar damage (Supplementary Figure 5).

*AagTg* mice, despite being exposed to a lower MMS dose, nevertheless experienced similar or increased MMS-induced cerebellar degeneration compared to WT mice (2036 ± 238 pyknotic nuclei in *AagTg* males versus 1332 ± 179 in WT males, p = 0.03; 1937 ± 254 in *AagTg* females versus 1753 ± 175 in WT females, p = 0.54). Veliparib treatment (10 mg/kg) almost completely rescued *AagTg* mice, regardless of gender (Figure 5). Olaparib, at the dose of 50 mg/kg, not only did not protect male or female *AagTg* mice, but, remarkably, increased the tissue damage in MMS-treated *AagTg* males while having little effect in females (2890 ± 251 in males, p = 0.03; 1632 ± 420 in females, p = 0.53; Figure 5). Administration of a much lower Olaparib dose (1 mg/kg) was able to significantly protect both male and female *AagTg* mice (Figure 5). Our data suggest that knowledge of PARP inhibitor characteristics, with respect to DNA trapping, and the use of appropriate doses are crucial in order to obtain the desired therapeutic efficacy.

*Parp1* gene deletion completely rescued MMS-induced cerebellar degeneration in both the WT and *AagTg* backgrounds, regardless of gender (Figures 4 and 5). Our findings demonstrate that: (i) PARP inhibitors can protect from MMS-induced cerebellar degeneration; (ii) as in the retina, unlike the *Parp1* deletion, the ability of PARP inhibitors to protect against MMS-induced AAG-dependent cerebellar degeneration depends on AAG levels and PARP inhibitor properties and doses.

## DISCUSSION

We observed a tissue specific sexual dimorphism after treatment with an alkylating agent. Specifically, MMS-treated WT female mice are partially protected against retinal degeneration, as compared with WT male mice, while both sexes exhibit similar cerebellar degeneration. Moreover, treatment of WT male mice with estrogen resulted in protection against MMS-induced retinal degeneration, providing an explanation for the relative resistance to alkylation-induced retinal degeneration in female mice. Gender differences in response to *Parp1* deletion or PARP inhibition were recently reported using mouse models of cerebral ischemia, endotoxemia and autoimmune nephritis, [46–50, 56]. In particular, it was demonstrated that (i) female mouse models are partially resistant to cerebral ischemia, endotoxemia and autoimmune nephritis; (ii) treatment of male mice with estradiol reduces PARP1 activation and exerts a protective effect against cerebral ischemia, endotoxemia and autoimmune nephritis [46, 47, 50]; (iii) in the absence of functional PARP1, male mice are preferentially protected against cerebral ischemia, endotoxemia and autoimmune nephritis compared to female mice, in which PARP1 deletion/inhibition offered no benefit in pathological outcome. PARP1 has been shown to interact with the estrogen receptor alpha (ERα) to form a complex that binds to DNA *in vitro* [46]. The binding of this ERα/PARP1 complex to DNA is enhanced by the presence of the estrogen ligand. Thus, it has been proposed that, in females, estrogen may anchor the PARP1/ERα complex to specific regions of DNA, reducing its availability to access and bind DNA SSBs, thus reducing PARP1 hyperactivation and cell death in the experimental models of disease. PARP1 was also shown to bind and PARylate ERα, leading to increased binding of ERα to the estrogen response element within the promoters of target genes, thus promoting ERα-mediated transcription [57]; estradiol enhances PARlylation of ERα and ERα-mediated transcription. Moreover, ERα is known to interact with other proteins involved in BER, namely AAG, FEN-1 and APE1 [58]. Binding of ERα with these proteins may influence not only ERα-mediated transcription, but also the expression or function of each protein. Expression of ERα has been reported in rodent and human photoreceptors [59, 60]. Together, these findings suggest that a direct interaction between BER proteins, PARP1 in particular, and estrogen receptors could lead to changes in DNA repair, PARP activation and/or gene expression. Such changes presumably underlie the sexual dimorphism of MMS-mediated retinal degeneration we have observed in mice. The known interaction between ERα and protein networks involved in cell fate determination and the oxidative stress response might also contribute to the sexual dimorphism [58]. The protection against alkylation-induced retinal degeneration that we observed in estrogen-treated male mice further supports the proposed model that ERα/estrogen and the PARP1/BER pathways interact, and also confirms the role of estrogen in protecting against alkylation-induced retinal degeneration.

In contrast to the retina, cerebellar degeneration induced by MMS in WT mice was not sexually dimorphic. The fact that ERα expression is lower or absent in the cerebellum [61, 62] might explain the lack of sexual dimorphism for cerebellar degeneration in MMS-treated WT mice. The *AagTg* mice show increased MMS-induced retinal and cerebellar degeneration compared to WT mice, as seen previously [35, 36]. Note that we did not observe any sexual dimorphism in the retinas of MMS-treated *AagTg* mice; this may be due to the inability of ERα to compensate for PARP1 hyperactivation due to the higher expression of AAG. In addition, it has been shown that interaction between AAG and ERα causes decreased ERα-mediated transcription and increased AAG catalytic activity [63]. Excessive suppression of ERα-mediated transcription in *AagTg* mice might further contribute to the lack of sexual dimorphism in the retinas of *AagTg* mice. Overall, our data underscore recent arguments for investigating and understanding sex differences in both basic and clinical science [64–67]. Furthermore, the sexually dimorphic response to alkylation-induced damage underscores the importance for sexual-stratification in cancer chemotherapy trials, especially those that utilize alkylating agents.

We found that *Parp1* gene deletion resulted in complete protection against MMS-induced retinal and cerebellar degeneration in both WT and *AagTg* mice, regardless of gender. It has been described previously that female and male mice exhibit different responses to *Parp1* deletion in other mouse models of disease. Indeed, *Parp1* deletion has been shown to protect male, but not female, animals against stroke and autoimmune-nephritis [47–50, 68]. A sex-specific bias for distinct cell death pathways in some tissues might account for the sexually dimorphic response to *Parp1* deletion observed in these experimental models. It appears that, upon specific kinds of stress, female cells preferentially activate an apoptotic pathway while male cells favor a PARP1-dependent necrotic pathway [69–71]. It is well known that DNA alkylating agents can induce necrosis in a PARP1-dependent manner, although this is likely to be cell type-dependent [72–74]. Even though female mice are partially protected against alkylation-induced retinal degeneration compared to male mice, *Parp1* gene deletion completely rescued both males and females against alkylation-induced retinal and cerebellar degeneration. We hypothesized that alkylation treatment induces retinal and cerebellar degeneration via the PARP1-dependent necrotic pathway in both males and females, but that female photoreceptors are just more resistant to such necrosis.

We found that treatment with PARP inhibitors also protects against MMS-induced AAG-dependent neuronal damage, but their efficacy depends mainly on their chemistry and dosage. Indeed, Veliparib, at a dose of 10 mg/kg, was able to protect against MMS-induced retinal and cerebellar degeneration in both WT and *AagTg* mice regardless of the gender, whereas Olaparib, at a dose of 50 mg/kg, was able to significantly protect only *AagTg* mice from retinal degeneration and WT male mice from cerebellar degeneration. Moreover, the extent of protection after Olaparib treatment was consistently lower than that observed after Veliparib treatment. It has been shown that, in addition to catalytic inhibition, some PARP inhibitors can induce cytotoxic PARP-DNA complexes, most likely by enhancing the crosslink between PARP and AP sites, or by inducing a PARP conformational change that traps PARP at the site of damage [39, 41, 44, 75]. Such trapping would interfere with DNA repair, replication and transcription. The formation of such a toxic complex appears to be a suicidal event when BER is overwhelmed, as can be the case with MMS treatment [41–43]. The potency in trapping PARP differs markedly among PARP inhibitors and it is not correlated with the catalytic inhibitory properties of each drug [39]. Therefore, PARP inhibitors can produce two competing effects: (i) resistance to MMS-induced cell death by PARP catalytic inhibition that prevents NAD^+^/ATP depletion, or (ii) sensitivity to MMS-induced necrotic cell death by trapping of PARP on DNA. Even though Olaparib has a higher catalytic inhibition activity, it also has much higher PARP-DNA trapping activity compared to Veliparib, thus making Olaparib a more potent cytotoxic compound than Veliparib [39]. As previously stated, in contrast to Veliparib, we observed absent or reduced Olaparib protection against MMS-induced neuronal degeneration, which may be due to Olaparib’s higher PARP-DNA trapping activity. Notably, Olaparib treatment (50 mg/kg) resulted in increased, rather than decreased, MMS-induced cerebellar degeneration in *AagTg* male mice. We hypothesize that the increased AAG activity in *AagTg mice* might lead to many more AP sites and SSBs and, thus, more substrate for Olaparib PARP-DNA trapping activity. Administration of a much lower dose of Olaparib (1 mg/kg) in *AagTg* mice resulted in protection against MMS-induced cerebellar degeneration in both males and females, supporting our hypothesis and suggesting that knowledge of PARP inhibitor properties and use of appropriate dosages is necessary to obtain therapeutic efficacy. Even so, protection against MMS-induced cerebellar degeneration after treatment with low doses of Olaparib (1 mg/kg) was still not at the level observed after Veliparb treatment (10 mg/kg) further suggesting that PARP inhibitors with less trapping activity, such as Veliparib, are more efficacious at preventing alkylation-induced neuronal degeneration. Finally, an additional dose of PARP inhibitors 24 hours after MMS treatment did not result in increased efficacy for either compound and, notably, in the case of Veliparib, resulted in reduced protection against retinal degeneration in female *AagTg* mice, suggesting that a single dose pre-MMS treatment is sufficient for therapeutic efficacy and additional doses may only cause increased damage due to PARP-DNA trapping activity.

In addition to protecting against neuronal degeneration, PARP inhibitors also protect *AagTg* mice against alkylation-induced body weight loss, regardless of the gender. We have shown that AAG mediates MMS-induced whole-animal lethality accompanied by cytotoxicity in organs other than retina and cerebellum [35]. Therefore, PARP inhibitors might also exert a protective effect in several other organs resulting in reduced overall weight loss.

Taken together, our data show that low doses of PARP inhibitors, or PARP inhibitors with less PARP-DNA trapping ability, can protect against alkylation induced neuronal degeneration. Alkylating agents are commonly used in cancer therapy. It is possible that the alkylation-induced neuronal degeneration and organ toxicity we observed in mice [35, 36] could be a rate-limiting side effect of alkylating agents used in cancer chemotherapy. Given the wide inter-individual variability of AAG [35, 51, 52] and PARP1 activity [76] in human populations and the sexual dimorphism we observed, toxicity might be limited to particular subpopulations, making it difficult to generalize. Nevertheless, our findings in mice may potentially prove to have important clinical relevance.

PARP inhibitors are used in a clinical setting for the treatment of various tumors, either alone or in combination with other chemotherapeutics, including alkylating agents [37, 38]. PARP inhibitors allow selective killing of cancer cells because the trapping/cytotoxic effect makes use of deficiencies in DNA repair systems that are unique to individual types of cancer (e.g. BRCA1/BRCA2-deficient tumors), as compared with normal tissue [40, 77–79]. PARP inhibitor cytotoxicity is enhanced when they are combined with alkylating agents [41–43]. Therefore, the recent surge in the use of PARP inhibitors in combination with alkylating agents might represent a powerful tool for obtaining better therapeutic efficacy in cancer treatment, while avoiding the collateral effects of alkylating agents in healthy tissues of the subpopulations mentioned above. Moreover, our findings suggest that low doses of PARP inhibitors, or PARP inhibitors with less DNA trapping ability, might also be used to treat neurodegenerative disorders that involve PARP1 activation [80, 81], and that it would be worthwhile to identify PARP inhibitors with little or no PARP-DNA trapping activity.

## MATERIALS AND METHODS

### Animals and treatments

*Aag* transgenic (*AagTg*) mice were described previously [35, 36, 82]. *Parp1^−/−^* mice were purchased from Jackson Laboratories [83] and backcrossed to C57BL/6. WT and *AagTg* mice were on a C57BL/6 genetic background. *Parp1^−/−^* and *AagTg*/*Parp1^−/−^*mice were on mixed genetic background (C57BL/6:129S). All animal procedures were performed according the NIH guide for the Care and Use of Laboratory Animals.

MMS was injected intraperitoneally. Veliparib (Selleck Chemichals Inc) was administered by oral gavage (10 mg/kg) and Olaparib (Selleck Chemicals Inc) by intraperitoneal injections (50 mg/kg or 1 mg/kg). PARP inhibitor doses were chosen based on datasheet guidelines and previously published data [84, 85]. 17-β estradiol (E2, Sigma) was injected intraperitoneally. For experiments in the retina, where indicated, mice received daily E2 doses of 50 μg/kg.

### Histological analysis

Eyes were fixed in Carnoy’s fixative and brains in 10% formalin. Tissues were processed by the Histology Core Facility at the David H. Koch Institute for Integrative Cancer Research (MIT); they were paraffin-embedded, sectioned at 5 μm, and stained with hematoxylin and eosin (H&E). All H&E-stained slides were blindly analyzed using a Nikon Eclipse 6800 microscope, a Retiga Exi camera, Velocity and ImageJ softwares.

To quantify retinal degeneration, the number of rows of photoreceptor nuclei in the outer nuclear layer (ONL) of each eye was counted. A minimum of three sections, close to the optic nerve area, was analyzed for each eye. For each section, the number of row of photoreceptor nuclei in the ONL was counted at three different points along the retina and averaged. The averaged counts for each of the three sections were then averaged for each eye. All eyes in an experimental group were then averaged and standard errors calculated. To quantify cerebellar degeneration, images representative of each lobe in the cerebellum were taken. For each cerebellum, the number of pyknotic nuclei/mm^2^ in each lobe was counted and then averaged. The counts from each group were then averaged, and standard errors calculated.

### Statistics

Statistical analyses were performed using GraphPad Prism software. Statistical significance was determined using unpaired t-test. A p-value is considered significant if less than 0.05.

## SUPPLEMENTARY MATERIALS FIGURES



## Abbreviations

*O*^6^meG: *O*^6^-methylguanine
7meG: 7-methylguanine
3meA: 3-methyladenine
BER: base excision repair pathway
AAG: alkyladenine DNA glycosylase
APE1: apurinic/apyrimidinic endonuclease
SSB: DNA single-strand break
Pol Δ: DNA polymerase Δ
5’dRP: 5’deoxyribose-5-phosphate termini
PARP1: poly(ADP-ribose) polymerase 1
NAD+: nicotinamide adenine dinucleotide
PAR: polymers of ADP-ribose
XRCC1: X-ray repair cross-complementing protein 1
ATP: adenosine triphosphate
MMS: methyl methanesulfonate
DSB: DNA double-strand break
E2: 17-Δ estradiol
ERα: estrogen receptor alpha
FEN-1: flap endonuclease 1
