# Supplementary material for: PARP inhibitors protect against sex- and AAG-dependent alkylation-induced neural degeneration
Source: Oncotarget. 2017 Aug 3;8(40):68707–20. doi: 10.18632/oncotarget.19844 (PMC5620290; doi:10.18632/oncotarget.19844)
Supplement: Supplementary file 1 [file oncotarget-08-68707-s001.pdf]

PARP inhibitors protect against sex- and AAG-dependent alkylation-induced neural degeneration

SUPPLEMENTARY MATERIALS

Aag expression analysis

RNA was extracted from retinas using RNeasy Mini Kit (Qiagen) and first-strand cDNA was synthesized using The SuperScript® III First-Strand Synthesis System (Invitrogen), according to the manufacturer's instructions. Quantitative real-time PCR was performed using 7500 Fast Real-Time PCR machine (Applied Biosystems) with Taqman primers and probe (Invitrogen).

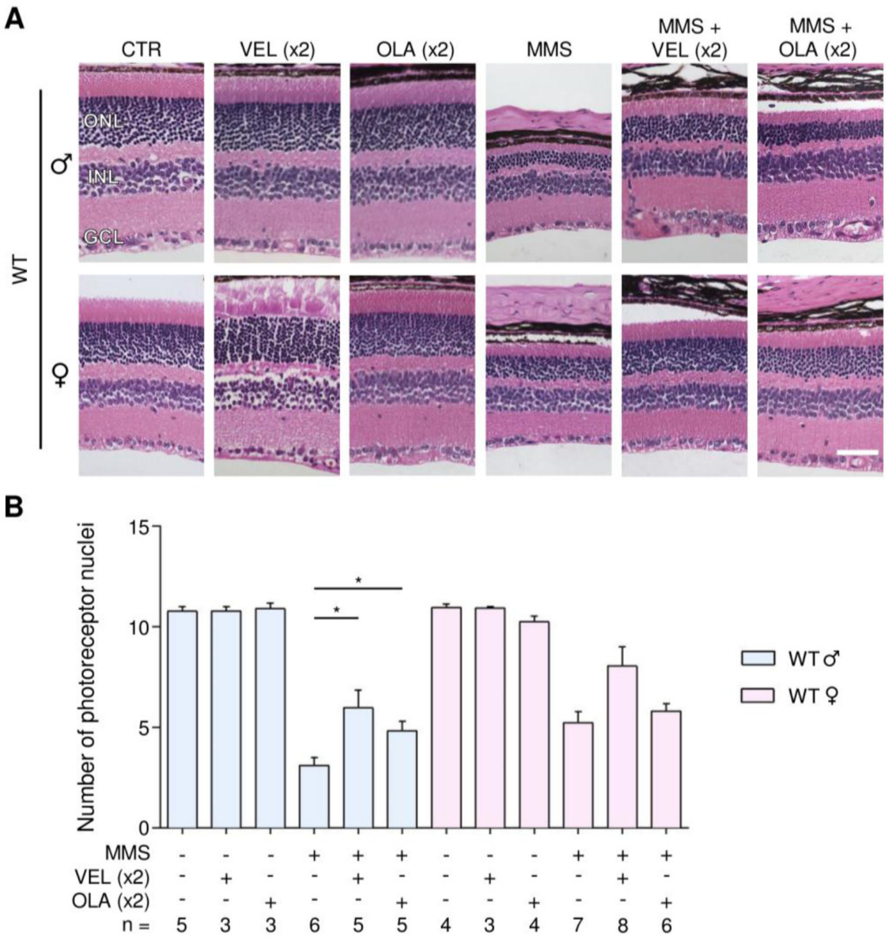

Supplementary Figure 1: Combined pre- and post-MMS treatment with PARP inhibitors does not increase protection against sex- and AAG-dependent MMS-induced retinal degeneration in WT mice. (A) Representative H&E-stained images of retinas from WT mice 7 days after MMS (75 mg/kg) and/or PARP inhibitor (VEL, Veliparib 10 mg/kg; OLA, Olaparib 50 mg/kg) treatment, as indicated. PARP inhibitors were administered 1 hour prior to and 24 hours (x2) after MMS treatment. Magnification is 200X (scale bar 50 μm); ONL, outer nuclear layer; INL, inner nuclear layer; GCL, ganglion cell layer; CTR, untreated control mice; ♂, males; ♀, females. Representative images for "CTR" and "MMS" injected groups are the same as in Figure 1. **(B)** Quantification of rows of photoreceptor nuclei in the outer nuclear layer of WT mice 7 days after MMS (75 mg/kg) and PARP inhibitor (VEL, Veliparib 10 mg/kg; OLA, Olaparib 50 mg/kg) treatment, as indicated; *, $p < 0.05$; ♂, males; ♀, females.

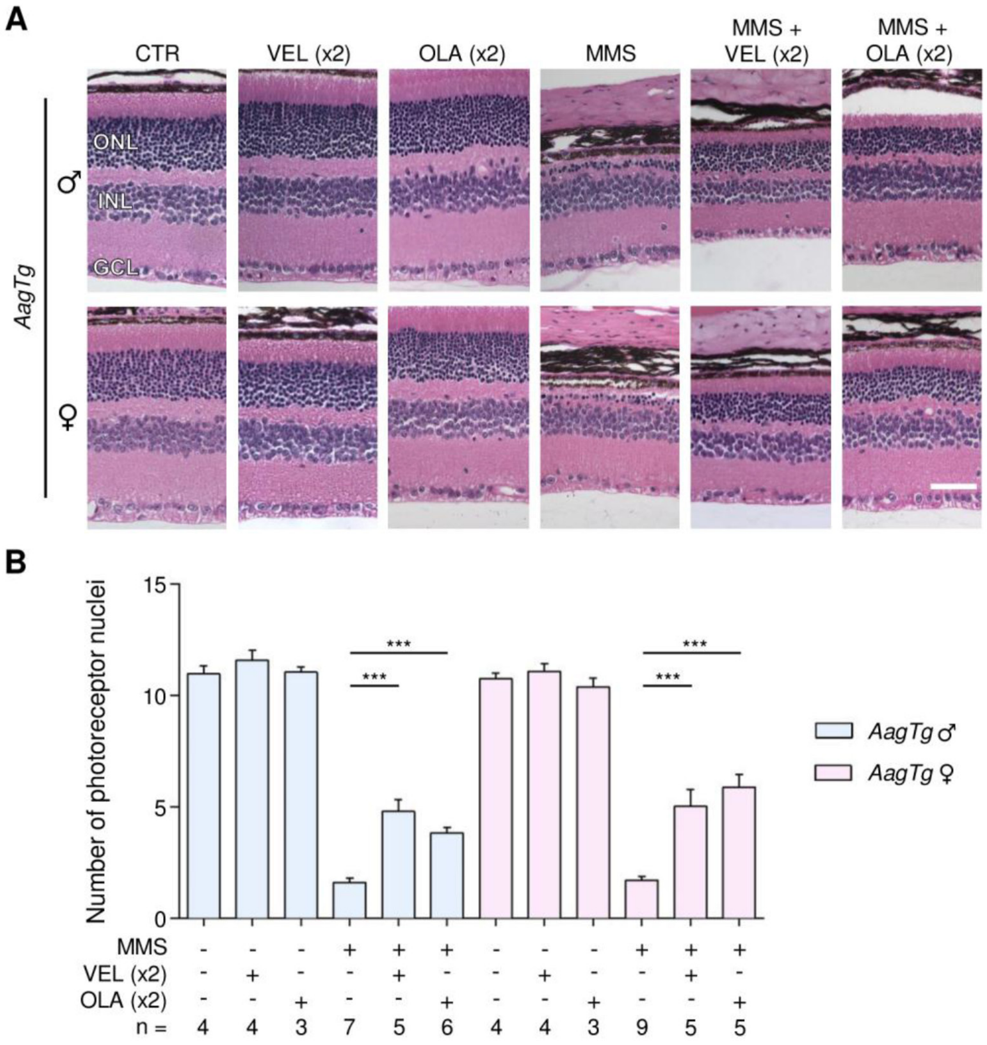

Supplementary Figure 2: Combined pre- and post-MMS treatment with PARP inhibitors does not increase protection against AAG-dependent MMS-induced retinal degeneration in *AagTg* mice. (A) Representative H&E-stained images of retinas from *AagTg* mice 7 days after MMS (75 mg/kg) and/or PARP inhibitor (VEL, Veliparib 10 mg/kg; OLA, Olaparib 50 mg/kg) treatment, as indicated. PARP inhibitors were administered 1 hour prior to and 24 hours (x2) after MMS treatment. Magnification is 200X (scale bar 50 µm); ONL, outer nuclear layer; INL, inner nuclear layer; GCL, ganglion cell layer; CTR, uninjected control mice; ♂, males; ♀, females. Representative images for "CTR" and "MMS" injected groups are the same as in Figure 2. **(B)** Quantification of rows of photoreceptor nuclei in the outer nuclear layer of *AagTg* mice 7 days after MMS (75 mg/kg) and PARP inhibitor (VEL, Veliparib 10 mg/kg; OLA, Olaparib 50 mg/kg) treatment, as indicated. ***, $p < 0.001$; ♂, males; ♀, females.

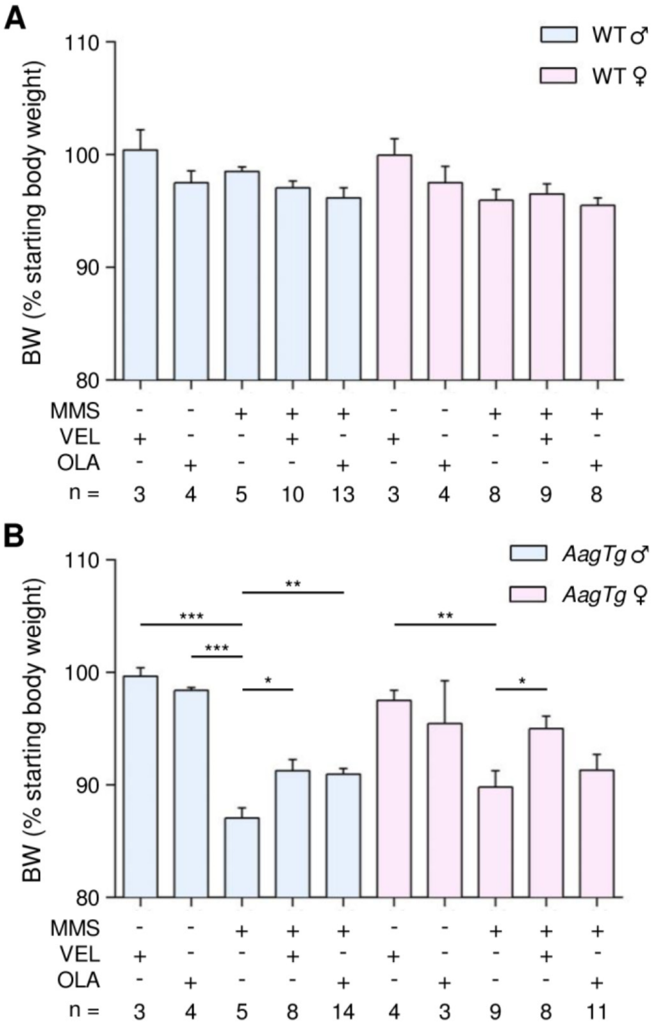

Supplementary Figure 3: PARP inhibitors protect *AagTg* mice from MMS-induced weight loss. Body weight (BW), as percentage of the initial body weight, 24 hours following MMS (75 mg/kg) and PARP inhibitor (VEL, Veliparib 10 mg/kg; OLA, Olaparib 50 mg/kg) treatment, is illustrated for WT **(A)** and *AagTg* **(B)** mice. PARP inhibitors were administered 1 hour prior to MMS-treatment. *, $p < 0.05$; **, $p < 0.01$; ***, $p < 0.001$; ♂, males; ♀, females.

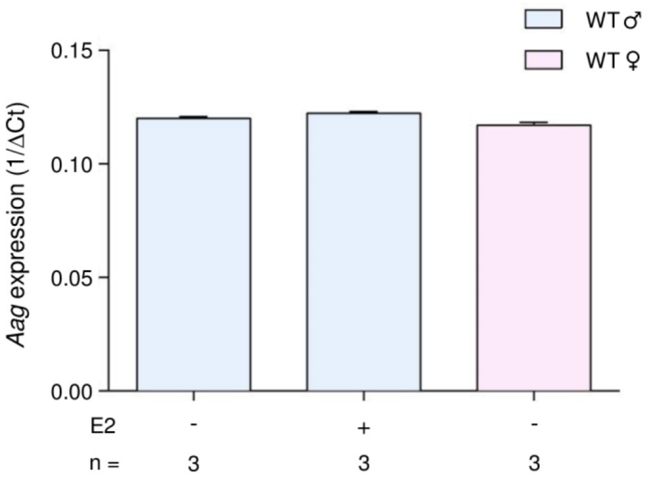

Supplementary Figure 4: *Aag* expression levels are not affected by sex. Quantitative real-time PCR analysis for *Aag* in the retinas of WT male and female mice uninjected or injected with 17-ß estradiol (E2, 50 µg/kg/day) as indicated. E2 was injected 1 time/day for 2 days. Retinas were harvested 2 hours post-injection at day 2. *Aag* expression levels are expressed as 1/ΔCt.

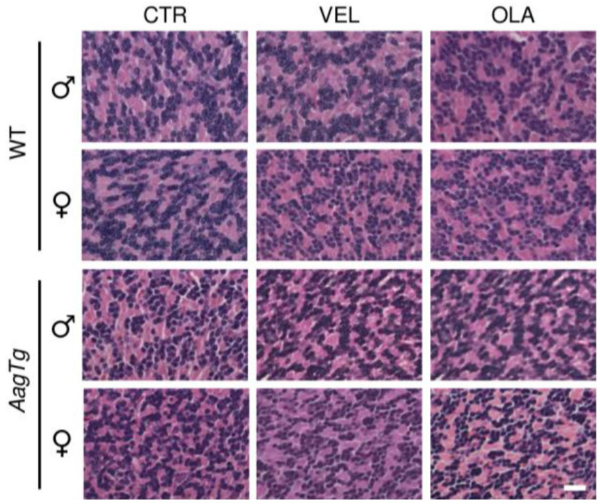

Supplementary Figure 5: Administration of PARP inhibitors themselves do not induce cerebellar degeneration. Representative H&E-stained images of cerebellar granule cells from WT and *AagTg* mice uninjected (CTR) or injected with either Veliparib (VEL, 10 mg/kg) or Olaparib (OLA 50 mg/kg). Cerebella were harvested 7 hours after treatment with PARP inhibitors. Magnification is 600X (scale bar 20 µm). Representative images for "CTR" group are the same as in Figures 4 and 5.
